# The Environmental Analysis of the Post-Use Management Scenarios of the Heat-Shrinkable Film

**DOI:** 10.3390/polym17050690

**Published:** 2025-03-05

**Authors:** Patrycja Walichnowska, Józef Flizikowski, Andrzej Tomporowski, Marek Opielak, Wojciech Cieślik

**Affiliations:** 1 Faculty of Mechanical Engineering, Bydgoszcz University of Science and Technology, Kaliskiego 7, 85-796 Bydgoszcz, Poland; jozef.flizikowski@pbs.edu.pl (J.F.); andrzej.tomporowski@pbs.edu.pl (A.T.); 2 Faculty of Transport and Informatic, University of Economics and Innovation in Lublin (WSEI), Projektowa 4, 20-209 Lublin, Poland; m.opielak@pollub.pl; 3 Faculty of Civil and Transport Engineering, Poznan University of Technology, Piotrowo 3, 60-965 Poznan, Poland; wojciech.cieslik@put.poznan.pl

**Keywords:** LCA, polymer film, post-use management, recycling

## Abstract

The post-use management of plastic films, including shrink films, poses a significant environmental and technological challenge for the industry. Due to their durability and difficulty in degradation, these wastes contribute to environmental pollution, generating microplastics and greenhouse gas emissions during improper disposal. This paper examines different post-use management methods for shrink wrap, such as recycling, landfilling, and incineration, and assesses their impact on the environmental impact of the bottle packaging process using a life-cycle analysis (LCA). This study shows that the recycling option has the lowest potential environmental impact. Compared to other post-use management options, recycling reduces the potential environmental impact by more than 50%. The analysis also shows that the tested scenario using recycled film and photovoltaic energy has the lowest potential environmental impact. Using recycled film and powering the process with renewable energy reduces the potential environmental impact by about 95% compared to Scenario 1 and by about 85% in Scenario 3.

## 1. Introduction

Plastics are very important in modern industry and everyday life thanks to their user-tailored functional properties. They are characterized by lightness, mechanical strength, corrosion resistance and the ability to adapt their properties to specific applications. All these features mean that plastics are used in various fields, from the automotive and construction industries to electronics and medicine. At the same time, the above-mentioned features that make plastics so widely used lead to the problem of increasing waste and the challenge of implementing a sustainable economy [1]. According to Zepa et al. [2], the European Union produces around 26 million tonnes of plastic waste, and only 30% of that is recycled. This is a serious problem, as plastics can break down under the impact of ultraviolet light, releasing microplastics into the environment, which have a negative impact on human health and the environment [3,4,5]. Plastics are widely used in the packaging industry, where a commonly used material facilitating transport is thermo-shrinkable films, which are flexible, lightweight and recyclable [6,7]. Their low bulk density and small thickness mean that, despite a number of advantages, films of this type, compared to rigid plastics, pose a significant challenge for recycling processes in economic and operational terms [8,9]. The circular economy model implemented in Europe concerns the reuse of plastic waste, which ceases to be a problem and becomes a valuable material in the packaging industry, among others. Research is important in terms of selecting the appropriate technology for processing film materials and additives to obtain post-recycling materials with similar functional properties to the product before processing [10].

Plastic film waste can be managed in various ways using technologies and methods adapted to the type and degree of contamination of the material. One example of management is recycling, i.e., the process of processing waste to recover raw materials or materials that can be reused to produce new products. The recycling of heat-shrinkable films can be divided into several types depending on the methods used and the degree of material processing. According to Ragaert et al. [11], in a circular economy, recycling materials are classified based on the product made from the secondary raw materials. In closed-loop recycling, recovered plastics are reused to produce the same product as the original. This can be made entirely from recycled material or mixed with new plastic, allowing for further recycling. Open-loop recycling, on the other hand, involves transforming recovered plastics into other products, such as PET bottles into textile fibers. However, reuse does not necessarily mean that the final product has a reduced value. There are several types of recycling. Mechanical recycling involves collecting, sorting, washing and mechanically processing the film, including shredding and melting it, which produces granulate that is used in the production of new products, such as films, bags or pipes. Chemical recycling, in turn, includes chemical decomposition processes, such as pyrolysis, which allow LDPE to be transformed into monomers or other chemical raw materials used to produce new materials. Chemical recycling allows for the recovery of high-quality raw materials that can be used in a wide range of industrial applications while minimizing the amount of waste. Waste plastics are pyrolyzed into liquid, gaseous, and solid products. Depending on the reaction conditions, slow, fast and flash pyrolysis are distinguished. Fast (10–200 °C/s) and flash (>1000 °C/s) pyrolysis require fine-grained raw materials and specialized equipment, which limits their industrial application [12,13]. Gasification can also be considered a chemical recycling method. Gasification is a process in which carbon-based fuels are converted into a gas with a calorific value. The resulting gas, called syngas, consists primarily of carbon monoxide (CO) and hydrogen (H_2_). It can be used to produce fuels, chemicals and even new plastics [14]. According to Vitale et al. [15], among the methods of gasification of plastics, gasification with air is the most used because the process of decomposing polymers requires significant amounts of energy. All methods, although they differ in technology and the intended use of the final products, have a common goal—to reduce the impact of film waste on the environment and create a more sustainable waste management system. Another method of waste management is waste storage, which involves collecting waste in appropriately prepared landfills, which are designed to minimize the impact on the environment. At the landfill, the waste is arranged in layers, compacted, and then covered with soil or other materials to limit its decomposition in contact with air and prevent the emission of unpleasant, strong odors. Although waste storage is a relatively cheap method, it is associated with problems such as greenhouse gas emissions, the occupation of large areas and the risk of environmental pollution. Another method of waste management is waste incineration, which involves controlled combustion at high temperatures in specially designed incinerators, which reduces the amount of waste and recovers energy in the form of heat or electricity [16,17,18,19].

Analyzing the structures of heat-shrinkable films available on the market, they can be divided into single-layer and multi-layer films. Single-layer films are commonly used to produce secondary packaging and in agriculture, while multi-layer films, which may consist of a dozen or so layers, are used as primary packaging. In the processes of mass packaging of beverage bottles, multi-layer films are used to enable the transport of a group of bottles to the recipient. According to Walichnowska et al. [10], the most energy-consuming stage of the packaging process is welding film. This process involves shrinking the film under the influence of heat in a chamber, thus creating a practical package. The process of thermal shrinking of the film requires the use of precisely controlled conditions, such as the appropriate temperature and time of exposure to heat, to ensure even adhesion of the film and avoid its damage. Although this stage is the most energy-consuming, it is also necessary to achieve high-quality packaging that meets the requirements of the logistics industry and consumers. The films used in this process are characterized by their high strength, flexibility and resistance to mechanical damage, protecting the packed bottles during transport and storage.

Research on the impact of the post-use management of plastic waste combined with LCAs can be found in many available scientific studies. Dong et al. [17] conducted a life-cycle analysis to assess the impact of advanced recycling of post-use plastics on greenhouse gas emissions, fossil energy consumption, water consumption and solid waste generation, focusing on pyrolysis as one of the main technologies for converting waste into new plastics. Liu et al. [20] examined the costs and environmental impacts of various waste management strategies. Their analysis showed that appropriate waste collection and further development of recycling technologies can help reduce the costs of the process and reduce its environmental impact. Ncube et al. [21] discussed the main aspects of food packaging waste management, focusing on its management in Africa, where it will pose a major problem in the future. The authors point out that more environmentally friendly solutions should be sought, models of its reduction should be adopted, and activities related to waste recycling should be implemented. Tomić et al. [22] conducted an LCA in which they analyzed waste management technologies. The studies showed that pyrolysis is characterized by potentially lower greenhouse gas emissions compared to incineration with energy production. The authors showed that this method of waste management meets EU directives and can help in the future to achieve the goal of a circular economy and reduce resource consumption. Xayachak et al. [23] conducted an LCA of three processing scenarios for polyethylene and polypropylene: landfill, pyrolysis and gasification. The authors showed that pyrolysis with monomer recovery, as part of a closed recycling loop, provides significantly greater environmental benefits compared to open systems.

The analysis of the current state of knowledge and technology has shown that the literature contains studies on the life cycle assessment (LCA) of heat-shrinkable films, especially in the context of various scenarios of their post-use management [24,25,26,27]. Previous studies focus mainly on technological or ecological aspects but do not consider the impact associated with the process of the mass packaging of bottles. Due to this fact, it is considered important to conduct research on the environmental analysis of the post-use management of the heat-shrinkable film used in the process of the mass packaging of bottles. This article aims to demonstrate which of the methods of post-use management of the film reduces the potential negative impact of the heat-shrinkable film used in the process of mass packaging. Additionally, the article examines how changing the type of film and powering the process with energy obtained from the Sun affects its potential impact on human health and ecosystem quality. The main limitations of the conducted research include, among others, different conditions of the conducted processes, management methods depending on local technologies or infrastructure. In addition, differences in the chemical composition of the heat-shrinkable film may affect the effectiveness of individual methods of the post-use management, including recycling, which may cause difficulties in comparing the results of other studies. A feature that distinguishes the conducted research from others is the fact that the life cycle of the selected heat-shrinkable film was focused on the process of mass packaging of bottles and the use of input data from a real conducted process for the research.

## 2. Materials and Methods

The functional properties of the film are crucial for the harmfulness of the process in terms of energy consumption, the emission of harmful substances and waste. The properties of the materials will be largely responsible for the functional properties of the film, the possible occurrence of deficiencies during production and, consequently the generation of waste and energy consumption to produce useless products. The paper will analyze variants of the packaging process using traditional film and film with the addition of reclaimed materials. In the article, Walichnowska et al. [10] tested the functional properties of heat-shrinkable film used in the process of mass packaging of bottles. This process was carried out in accordance with ASTM D2732 [28] free linear shrinkage tests. The tests were conducted using a Memmert machine with a bowl, along with silicone oil (Memmert GmbH + Co. KG, Schwabach, Germany). Tests were also carried out to determine the mechanical properties in the static tensile test in accordance with the PN-EN ISO 527-1:2020 [29] and PN-EN ISO 527-3:2019-01 [30] standards. The machine used in the test was TIRA test 27025 (TIRA GmbH, Schalkau, Germany). The tearing force of the tested film samples was also determined at a given length from the cut under specific load conditions (1600 g). These tests were carried out in accordance with the PN-EN ISO 6383-2:2005 standard [31]. The machine used was the Pro Tear Tearing Tester (Twing-Albert Instrument Company, New Berlin, NJ, USA). The results showed that the recycling film can be used instead of traditional film; therefore, we can continue research in scenarios using the new film and different ways to power machines.

To determine the impact of the post-use management of the film on the environment, a life cycle analysis (LCA) was conducted, which determines the impact of the selected product on the environment at each stage of its existence. The LCA is divided into four stages: the definition of the purpose and scope of the analysis, data analysis, conducting an environmental impact assessment and interpretation of the results [32,33,34]. When formulating the purpose and scope of the research, it was crucial to collect the most reliable and detailed information possible regarding the analyzed technical objects. This was achieved by establishing cooperation with a company involved in the production, distribution and sale of a wide range of food products.

**Figure 1:**
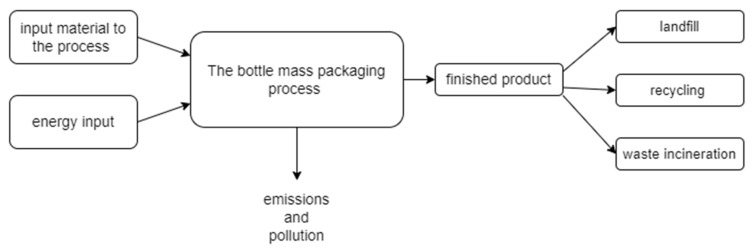
Analyzed technological process including the post-use management scenarios of the heat-shrinkable film (own elaboration).

To compare the potential environmental impacts of the process in question, an extended LCA was conducted considering waste management (Figure 1). This type of analysis does not end only with the output from the production unit but also considers the methods of waste management generated in the process, which affects the more comprehensive assessment of the environmental impact. Such studies aim to improve waste management while supporting sustainable development and efficient use of resources [35,36].

In conducting an LCA, it is important to collect data on the consumption of raw materials, energy and processes accompanying the tested object, including, e.g., transport. Table 1 presents the inventory data of the mass bottle packaging process, which was obtained from the company and determined for the assumed functional unit of 1000 packs. The presented input data refer to three process variants differing in the method of post-use management of the heat-shrinkable film. In all the variants tested, the process was powered by energy obtained from the country’s energy mix (Poland), in which 60% of energy comes from coal. As previously demonstrated by the research conducted [37], powering processes with energy from such a mix affects the deterioration of human health and ecosystems. In the study, based on information from the company, it was assumed that the film waste generated during packaging does not exceed 1% of the film used and is transferred in its entirety to the company responsible for collecting segregated waste. Due to limited access to data on the transport of packaging and subsequent film waste, it was assumed that the distribution and disposal of packaging takes place in one province in Poland. On this basis, it was assumed that a truck with a capacity of 24.7 tons travels approx. 100 km between the company, user and waste collection point, burning 27.6 l.

The research was conducted using SimaPro 9.6 software, specifically using the Ecoinvent v3 database available in it. This database contains diverse sets of data for different industry sectors, which allows for precise modeling of the product life cycle and comparison of different variants. Three different variants of film waste management were adopted as part of the research:

- Variant I: mechanical recycling.
- Variant II: waste incineration.
- Variant III: landfill.

Additionally, an analysis was also carried out to compare scenarios for the mass packaging of bottles in which polyethylene film was replaced with recycled film and energy from the country’s mix with energy from a photovoltaic installation to power the machines (without considering the method of film management). These scenarios were analyzed:

- Scenario 1: recycling film, energy from country’s energy mix.
- Scenario 2: recycling film energy from photovoltaic installations.
- Scenario 3: polyethylene film, energy from photovoltaic installations.

The environmental analysis of the process in question was carried out in accordance with the standards [38,39]; additionally, the IMPACT World+ Endpoint method was used, which allows for a comprehensive analysis of the impact of human activities on the environment. It is characterized by a wide range of impact categories, covering both short-term effects, lasting up to 100 years from the emission, and long-term effects, which can occur even after a century. This method considers various aspects of impact, such as climate change, toxicity, acidification of the environment, as well as loss of biodiversity. Thanks to the division into short-term and long-term damage, it allows for the precise identification of potential threats in different time perspectives. The use of IMPACT World+ promotes more conscious decision-making in production processes and planning actions for sustainable development in given enterprises [40,41,42].

## 3. Results

Using the Impact World + endpoint method, the potential impact on the environment was determined for various post-use management scenarios of the mass packaging process which used the heat-shrinkable film. Table 2 presents the results of the analysis divided into effects on human health (expressed in DALY—one DALY is equivalent to the loss of one year of life in full health, where the causes of this loss are attributed to premature death or disability) and ecosystem quality (expressed in PDF × m^2^ × year—Potentially Disappeared Fraction of species per square meter per year). The analysis showed that the impact categories that had the greatest potential impact in all three variants studied, for human health, are water availability, human health (Variant I: 0.00012 DALY, Variant II: 0.00017 DALY, Variant III: 0.00064 DALY) and climate change, human health, long term (Variant I: 0.000228 DALY, Variant II: 0.000589 DALY, Variant III: 0.000349 DALY); additionally, for ecosystem the variants studied for quality are freshwater ecotoxicity, long term (Variant I: 1520 PDF × m^2^ × year, Variant II: 1540 PDF × m^2^ × year, Variant III: 2490 PDF × m^2^ × year) and climate change, ecosystem quality, long term (Variant I: 50.2 PDF × m^2^ × year, Variant II: 129 PDF × m^2^ × year, Variant III: 76.8 PDF × m^2^ × year). All tested variants showed the lowest environmental impact on human health in the category of ozone layer depletion impact (Variant I: 5.56 × 10^−9^ DALY, Variant II: 4.31 × 10^−9^ DALY, Variant III: 4.40 × 10^−9^ DALY). The ozone layer plays a very important role in protecting human health from the negative effects of the Sun’s ultraviolet radiation. Very small damages within this category indicate that the analyzed variants emit a small amount of ozone-depleting substances. The lower values for Variants II and III may result from controlled combustion processes and the limited emission of volatile substances during waste storage. However, according to Duan et al. [43], although trace gas emissions constitute a small fraction of total landfill emissions, they remain problematic due to their harmful impact on the environment and human health. In all three variants, in the category of damage to ecosystems, the potentially smallest impact was observed around ionizing radiation (Variant I—1.90 × 10^−8^ PDF × m^2^ × year, Variant II—8.20 × 10^−9^ PDF × m^2^ × year, Variant III—1.02 × 10^−8^ PDF × m^2^ × year). The values in this category are exceptionally low, which indicates the minimal impact of the variants studied on the quality of ecosystems in terms of ionizing radiation emissions. Ionizing radiation, such as gamma, alpha or beta radiation, is one of the most harmful factors for living organisms because it can lead to genetic mutations, DNA damage and disruptions in the functioning of ecosystems [44,45]. The analysis carried out concerned the management of packaging films that are not radioactive waste. Therefore, the impact values are small.

In the above-mentioned categories of impact on human health, i.e., ionizing radiation, human health and ozone layer depletion, Variant I is not characterized by the smallest potential impact among the variants analyzed. In the remaining nine categories, it has the smallest potential impact. Analyzing the damage to ecosystem quality, Variant I does not characterize the lowest amount of the impact among all the variants analyzed, in the following categories: ionizing radiation, ecosystem quality (the lowest impact—Variant II—8.20 × 10^−9^ PDF × m^2^ × year); land transformation, biodiversity (the lowest impact—Variant II—3.77 PDF × m^2^ × year); land occupation, biodiversity (the lowest impact: Variant II—1.46 PDF × m^2^ × year); water availability, freshwater ecosystem (the lowest impact: Variant II—0.0067 PDF × m^2^ × year); water availability, terrestrial ecosystem (the lowest impact: Variant III—0.00439 PDF × m^2^ × year). In the case of the water availability, freshwater ecosystem category, the impact is greatest for Variant I because the mechanical recycling of polyethylene waste involves cleaning, washing and separation processes that require large amounts of water [46,47].

**Figure 2:**
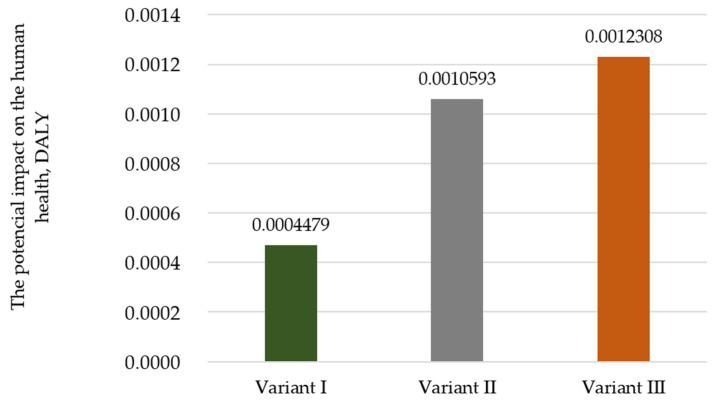
Impact of the analyzed variants on the human health, DALY (own elaboration).

The analyses also included a complete assessment of the impact of the studied variants on two areas of impact: human health and ecosystem quality. The results obtained allowed us to indicate which of the studied variants of using the film in the process of mass packaging and its post-consumer management is potentially the most harmful. The analysis showed that, for human health (Figure 2), the variant where the heat-shrinkable film is stored is characterized by the potentially greatest negative impact on the environment (Variant III—0.0012308 DALY); for the variant with the waste incineration, this impact is about 15% lower (Variant II—0.0010593 DALY) and, for the variant with recycling, it is about 60% lower (Variant I—0.0004479 DALY).

**Figure 3:**
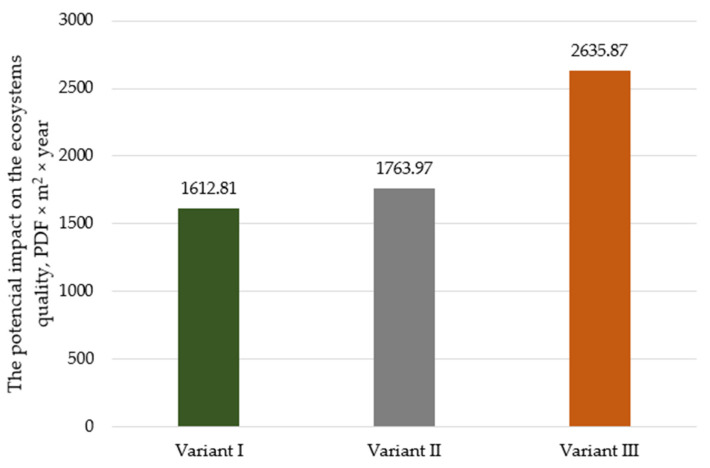
Impact of the analyzed variants on the ecosystem quality, PDF × m^2^ × year (own elaboration).

In the case of impacts on ecosystem quality (Figure 3), the potentially highest impact is noticeable for Variant III (2635.87 PDF × m^2^ × year). Using waste incineration (Variant II—1763.97 PDF × m^2^ × year) as a method of post-use management of the film, it reduces damage by approx. 34% and, in the case of recycling, by approx. 39% (Variant I—1612.81 PDF × m^2^ × year).

Additional analysis of the scenarios of the mass bottle packaging process in which the energy source and the type of heat-shrinkable film used were changed showed that all three scenarios studied in the human health area (Table 3) are characterized by the lowest value of the impact on ozone layer depletion (Scenario 1—7.50 × 10^−^^10^ DALY, Scenario 2—2.10 × 10^−^^10^ DALY, Scenario 3—4.32 × 10^−^^9^ DALY). Scenario 3 shows the highest impact values in almost all human health impact categories except human toxicity non-cancer (6.98 × 10^−^^6^ DALY), short term, where the highest value was shown for Scenario 1 (8.04 × 10^−^^6^ DALY). This is because, in Scenario 1, recycled film was used, which can contribute to the emission of toxic compounds from waste used in the production processes. Scenario 2 shows the lowest values in each category of impacts on human health. Considering the values of impacts on ecosystem quality, Scenario 3 also shows the highest impact values in each category. Scenario 2, apart from the land transformation category, shows the lowest impact values in all other impact categories. In this impact category, variants using energy from PV farms have higher values than Scenario 1, which results from the fact that photovoltaic farms occupy much larger areas than traditional power plants used in Scenario I in the country’s energy mix. As we can see in the impact on ecosystem quality, Scenario 3 shows higher impacts in all categories, which is related to the processes of producing primary polyethylene, which require intensive use of fossil raw materials, such as crude oil and natural gas. The extraction of these raw materials leads to the transformation of natural areas, which often results in the irreversible loss of habitats for many plant and animal species. Additionally, these processes generate soil and surface and groundwater pollution, which has a negative impact on local biodiversity, especially in the extraction areas.

**Figure 4:**
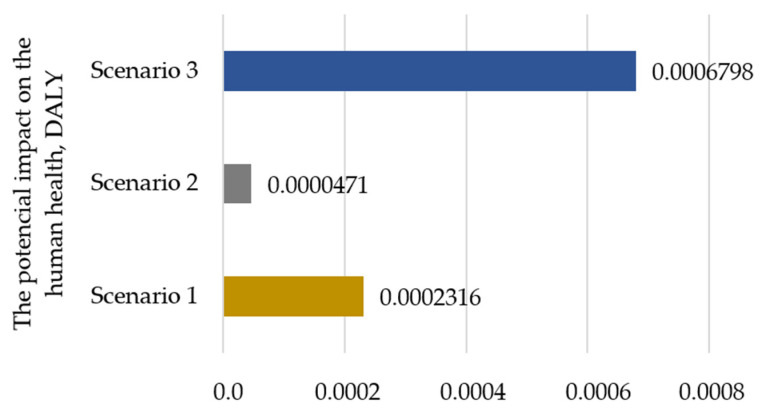
Impact of the analyzed scenarios on the human health, DALY (own elaboration).

The analysis also allowed for a comparison of the total impact of individual scenarios on human health and ecosystem quality. Figure 4 shows the impact of individual scenarios on human health. The scenario using recycled film and renewable energy to power the mass bottle packaging process is characterized by the lowest potential impact (Scenario 2—0.0000471DALY). Scenario 1 is characterized by an impact of 0.0001845 DALY higher, while Scenario 3 is characterised by 0.0006327 DALY.

**Figure 5:**
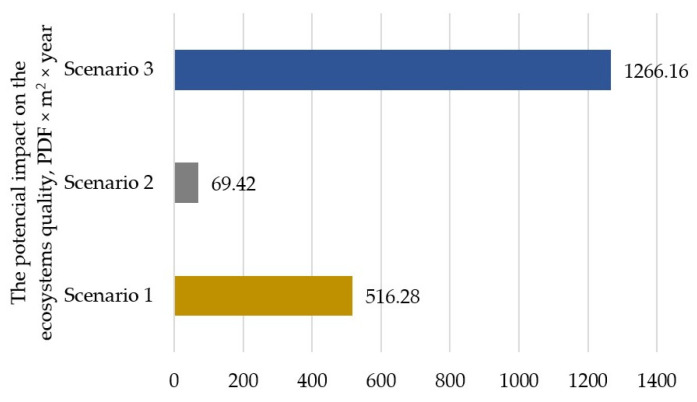
Impact of the analyzed scenarios on the ecosystem quality, PDF × m^2^ × year (own elaboration).

The conducted analysis also allowed for showing the potential impact on the ecosystem quality of the analyzed scenarios (Figure 5). Similarly to the human health, Scenario 2 is characterized by the lowest potential impact at the level 69.42 PDF × m^2^ × year. Scenario 1 is characterized by a potentially greater impact of 446.87 PDF × m^2^ × year compared to Scenario 2, while Scenario 3 is characterized by 1196.74 PDF × m^2^ × year.

## 4. Discussion

As the need to implement more ecological solutions in the plastics market grows, so does the need to provide data on their potential impact throughout the life cycle, with particular emphasis on post-use management [48]. The LCA of the heat-shrinkable film used in the process of mass packaging of bottles showed that the lowest potential impact on human health and ecosystem quality characterizes variant I (with recycling). Research by Hou et al. [49] also confirms that recycling plastic film waste provides environmental benefits instead of landfilling or incinerating it. Moreover, according to Björklund and Finnveden [50], among the waste management methods studied, recycling uses less energy and imposes less burden on the environment than landfilling or incineration. In the case of combustion of synthetic polymers, such as polyethylene terephthalate (PET) and polyethylene (PE), harmful substances are released into the atmosphere. The process produces volatile organic compounds, smoke, and particulate matter that may contain heavy metals [51].

Studies have also shown that the post-consumer waste management option in the form of incineration has a potentially smaller impact on the environment than landfilling. This is mainly due to the efficiency of incineration in reducing the amount of waste and limiting its long-term presence in the environment, which can minimize the emission of pollutants associated with their long-term decomposition. However, it should be emphasized that the incineration process is associated with the emission of greenhouse gasses and the potential release of toxic substances into the atmosphere, which pose a threat to the environment. Although incineration can be beneficial in certain aspects, e.g., in heat recovery, it is necessary to continue research on its potential impact on the environment [48,52,53]. According to Horodytska et al. [9], waste incineration with energy recovery should only be used when the waste is not suitable for recycling.

The main assumption of the circular economy is to preserve resources in a closed cycle of use [54,55]. Waste incineration, although studies have shown it to be an ecologically more beneficial solution compared to landfill, still raises some doubts in the context of this strategy. On the other hand, it should be pointed out that this method of management contributes to meeting energy needs and reducing the amount of waste [56,57]. Mechanical recycling, which includes collecting, sorting, washing and shredding waste, is currently the most popular type of recycling. Depending on the origin and composition of the waste, the process can proceed in different ways, e.g., for industrial waste, which is usually clean and free of organic contaminants, the recycling process is relatively simple and effective because its composition is well known. However, in the case of post-use waste, which is often a mixture of different polymers and contains numerous organic and inorganic contaminants, the process becomes much more complicated. Such waste requires more advanced sorting and additional purification steps, which is a challenge both technologically and economically [8,11,58,59]. The transformation towards a circular economy, as documented by Ferdous et al. [57], not only brings long-term environmental, social and economic benefits but also helps to protect natural resources and create new jobs.

The analysis of scenarios (which concerned the change in the type of film and the energy supplying the mass packaging process) showed that Scenario 2 is characterized by the lowest potential impact on human health and ecosystem quality. In this scenario, recycled film and energy obtained from a renewable source were used to pack bottles. The low values of impacts on the environment result from the use of recycled film, which reduces the demand for primary raw materials, which are associated with the extraction and processing of materials. The recycling process, although it generates emissions of undesirable gasses compared to the production of primary raw materials, is characterized by a lower amount of these emissions. Additionally, the use of renewable energy, from a photovoltaic farm, contributes to the reduction in greenhouse gas emissions, which, in large quantities, enter the air during the production of energy in conventional sources [60,61,62]. Studies have shown that implementing recycled film in the bottle packaging process and powering the processes with energy obtained from renewable sources reduces the negative impact and contributes to sustainable development. Such solutions additionally support the circular economy, reducing the amount of film waste ending up in the environment. Additionally, implementing photovoltaic installations in companies affects not only access to cheap and ecological energy but also provides independence and improves the company’s image.

The amount of waste worldwide will continue to grow due to increased consumption and economic development. The growing wealth of society in some parts of the world directly contributes to the growing demand for plastic packaging, which, after fulfilling its basic function, becomes waste. The situation in developing countries is particularly worrying, where the infrastructure for waste management does not keep up with the dynamic growth in its amount, which leads to problems related to illegal storage and environmental pollution. In Europe, further targets and regulations for plastic waste management are systematically introduced. Ambitious assumptions have been adopted, according to which the recycling of plastic packaging is to reach 50% by 2025 and increase to 55% by 2030. In addition, bans on the use of plastic bags and regulations limiting the use of polystyrene have been introduced with the aim of reducing the amount of waste and protecting the environment [20].

## 5. Conclusions

Plastic film waste is one of the most serious environmental challenges on a global scale, mainly due to its long-term impact on ecosystems. The plastics from which the films are produced are highly resistant to biodegradation processes, which results in their accumulation in the environment for hundreds or even thousands of years. During this time, they can lead to the pollution of various components of the environment, including soil, surface and ground water, and the atmosphere, by releasing harmful substances during physical and chemical degradation. The research conducted in the article has shown the following:

- Plastic recycling plays a key role in reducing the accidental or intentional release of polymers into the environment, thus contributing to reducing pollution;
- Of the three waste management methods analyzed—recycling, incineration with energy recovery and landfill—recycling has the lowest potential negative impact on the environment;
- Implementing recycling in waste management not only reduces the negative effects on the environment and human health compared to other waste treatment methods but also supports the circular economy;
- The process of waste incineration with energy recovery has about 15% less impact on human health and 34% less impact on the quality of ecosystems compared to landfill;
- Considering the sum of the impacts on human health and ecosystems, Variant I has the least potentially harmful impact of the analyzed options;
- The use of recycling as a method of managing film waste from mass packaging of bottles reduces the negative impact on the environment within the analyzed technical system;
- Recycling in this case reduces emissions, reduces the use of primary raw materials and supports the idea of a circular economy, limiting the negative effects on human health and the quality of ecosystems;
- Production of films from recycled raw materials and the use of renewable energy in the production process help to reduce the potential impact on human health and ecosystems;
- Scenario 3, despite the use of renewable energy, has the greatest impact on ecosystems, which results from the production of virgin polyethylene. This process requires greater use of natural resources and land occupation, leading to more intensive environmental degradation compared to scenarios using recycled materials.

Taking the above into account, it is recommended to continue research on methods of managing film waste to develop solutions that will not only reduce the negative impact of this waste on the environment but will also enable the real implementation of the principles of the circular economy on a wider scale, going beyond local conditions. It should be noted that one of the main limitations of the conducted research was its reference to a specific region, which means that these results may not be reflected in other regions, where environmental conditions, recycling infrastructure or legal regulations may differ.

## Figures and Tables

**Table 1:** Input data of the tested process (own elaboration).

Parameter	Variant I	Variant II	Variant III
Energy, kWh	45	45	45
Polyethylene film, kg	30	30	30
Fuel consumption, L/tkm	0.011	0.011	0.011
Post-use management scenario	mechanical recycling	waste incineration	landfill

**Table 2:** Results of environmental analysis of heat-shrinkable film used in the process of mass packaging of bottles for three analyzed variants of the post-use management scenarios (own elaboration).

Impact Category	Unit	Variant I	Variant II	Variant III
Climate change, human health, short term	DALY	0.0000809	0.000189	0.000136
Climate change, human health, long term	DALY	0.000228	0.000589	0.000349
Photochemical oxidant formation	DALY	8.03 × 10^−9^	2.30 × 10^−8^	2.47 × 10^−8^
Ionizing radiation, human health	DALY	2.92 × 10^−7^	1.15 × 10^−7^	1.44 × 10^−7^
Ozone layer depletion	DALY	5.56 × 10^−9^	4.31 × 10^−9^	4.40 × 10^−9^
Human toxicity cancer, short term	DALY	8.39 × 10^−6^	1.08 × 10^−5^	1.06 × 10^−5^
Human toxicity cancer, long term	DALY	4.65 × 10^−7^	8.27 × 10^−7^	8.92 × 10^−7^
Human toxicity non-cancer, short term	DALY	1.10 × 10^−5^	1.50 × 10^−5^	1.37 × 10^−5^
Human toxicity non-cancer, long term	DALY	1.31 × 10^−5^	2.33 × 10^−5^	2.49 × 10^−5^
Particulate matter formation	DALY	8.40 × 10^−6^	6.22 × 10^−5^	5.75 × 10^−5^
Water availability, human health	DALY	0.00012	0.00017	0.00064
Climate change, ecosystem quality, short term	PDF × m^2^ × year	17.50	40.80	29.50
Climate change, ecosystem quality, long term	PDF × m^2^ × year	50.20	129.00	76.80
Marine acidification, short term	PDF × m^2^ × year	1.30	3.38	1.99
Marine acidification, long term	PDF × m^2^ × year	12.00	31.10	18.30
Freshwater ecotoxicity, short term	PDF × m^2^ × year	6.17	6.16	5.43
Freshwater ecotoxicity, long term	PDF × m^2^ × year	1520.00	1540.00	2490.00
Freshwater acidification	PDF × m^2^ × year	0.0139	1.0900	1.0700
Terrestrial acidification	PDF × m^2^ × year	0.11000	7.0400	6.920
Freshwater eutrophication	PDF × m^2^ × year	0.00728	0.0102	0.015
Marine eutrophication	PDF × m^2^ × year	0.02970	0.1080	0.440
Ionizing radiation, ecosystem quality	PDF × m^2^ × year	1.90 × 10^−8^	8.20 × 10^−9^	1.02 × 10^−8^
Land transformation, biodiversity	PDF × m^2^ × year	3.95	3.77	3.92
Land occupation, biodiversity	PDF × m^2^ × year	1.49	1.46	1.47
Water availability, freshwater ecosystem	PDF × m^2^ × year	0.03240	0.0067	0.00701
Water availability, terrestrial ecosystem	PDF × m^2^ × year	0.00498	0.0470	0.00439
Thermally polluted water	PDF × m^2^ × year	0.00053	0.00059	0.00063

**Table 3:** Results of environmental analysis of heat-shrinkable film used in the process of mass packaging of bottles for three analyzed scenarios (own elaboration).

Impact Category	Unit	Scenario 1	Scenario 2	Scenario 3
Climate change, human health, short term	DALY	4.06 × 10^−5^	7.56 × 10^−6^	9.86× 10^−5^
Climate change, human health, long term	DALY	1.31 × 10^−4^	2.31 × 10^−5^	2.77 × 10^−4^
Photochemical oxidant formation	DALY	5.54 × 10^−9^	1.50 × 10^−9^	2.38 × 10^−8^
Ionizing radiation, human health	DALY	3.80 × 10^−8^	6.92 × 10^−9^	1.26 × 10^−7^
Ozone layer depletion	DALY	7.50 × 10^−10^	2.10 × 10^−10^	4.32 × 10^−10^
Human toxicity cancer, short term	DALY	3.77 × 10^−6^	1.82 × 10^−6^	9.14 × 10^−6^
Human toxicity cancer, long term	DALY	4.40 × 10^−7^	4.03 × 10^−8^	4.84 × 10^−7^
Human toxicity non-cancer, short term	DALY	8.04 × 10^−6^	5.16 × 10^−7^	6.98 × 10^−6^
Human toxicity non-cancer, long term	DALY	1.16 × 10^−5^	1.19 × 10^−6^	1.40 × 10^−5^
Particulate matter formation	DALY	1.90 × 10^−5^	3.57 × 10^−6^	4.84 × 10^−5^
Water availability, human health	DALY	1.71 × 10^−5^	9.26 × 10^−6^	2.25 × 10^−4^
Climate change, ecosystem quality, short term	PDF × m^2^ × year	8.79	1.64	21.30
Climate change, ecosystem quality, long term	PDF × m^2^ × year	28.80	5.07	60.80
Marine acidification, short term	PDF × m^2^ × year	0.75	0.13	1.58
Marine acidification, long term	PDF × m^2^ × year	6.92	1.22	14.60
Freshwater ecotoxicity, short term	PDF × m^2^ × year	0.44	0.31	6.16
Freshwater ecotoxicity, long term	PDF × m^2^ × year	465.00	58.20	1150.00
Freshwater acidification	PDF × m^2^ × year	0.54400	0.0447	0.656
Terrestrial acidification	PDF × m^2^ × year	3.46000	0.3050	4.320
Freshwater eutrophication	PDF × m^2^ × year	0.00124	0.0008	0.011
Marine eutrophication	PDF × m^2^ × year	0.03260	0.0079	0.103
Ionizing radiation, ecosystem quality	PDF × m^2^ × year	2.46 × 10^−9^	4.74 × 10^−10^	9.23 × 10^−9^
Land transformation, biodiversity	PDF × m^2^ × year	0.86	2.17	5.42
Land occupation, biodiversity	PDF × m^2^ × year	0.68	0.32	1.20
Water availability, freshwater ecosystem	PDF × m^2^ × year	0.00086	0.0003	0.00615
Water availability, terrestrial ecosystem	PDF × m^2^ × year	0.00176	0.0002	0.00327
Thermally polluted water	PDF × m^2^ × year	0.00034	0.00001	0.00035

## Data Availability

The raw data supporting the conclusions of this article will be made available by the authors on request.
